# Embedded Vision Sensor Network for Planogram Maintenance in Retail Environments

**DOI:** 10.3390/s150921114

**Published:** 2015-08-27

**Authors:** Emanuele Frontoni, Adriano Mancini, Primo Zingaretti

**Affiliations:** Dipartimento di Ingegneria dell’Informazione (DII), Università Politecnica delle Marche, Via Brecce Bianche, Ancona I-60131, Italy; E-Mails: frontoni@dii.univpm.it (E.F.); mancini@dii.univpm.it (A.M.)

**Keywords:** planogram integrity, retail environments, embedded sensors, wireless sensor networks, computer vision

## Abstract

A planogram is a detailed visual map that establishes the position of the products in a retail store. It is designed to supply the best location of a product for suppliers to support an innovative merchandising approach, to increase sales and profits and to better manage the shelves. Deviating from the planogram defeats the purpose of any of these goals, and maintaining the integrity of the planogram becomes a fundamental aspect in retail operations. We propose an embedded system, mainly based on a smart camera, able to detect and to investigate the most important parameters in a retail store by identifying the differences with respect to an “approved” planogram. We propose a new solution that allows concentrating all the surveys and the useful measures on a limited number of devices in communication among them. These devices are simple, low cost and ready for immediate installation, providing an affordable and scalable solution to the problem of planogram maintenance. Moreover, over an Internet of Things (IoT) cloud-based architecture, the system supplies many additional data that are not concerning the planogram, e.g., out-of-shelf events, promptly notified through SMS and/or mail. The application of this project allows the realization of highly integrated systems, which are economical, complete and easy to use for a large number of users. Experimental results have proven that the system can efficiently calculate the deviation from a normal situation by comparing the base planogram image with the images grabbed.

## 1. Introduction

Tours of customers and visitors of a retail store have to be planned, beginning from the structure and design of the store to the location of products for sale. This is true either for small shops or large shopping centers. Decisions are made by someone working in retail operations [1], the area of retail concerned with the daily functions of stores. Retail operations implicate all of the work that store staff does to keep a retail store functioning. They involve different activities and aspects, such as costumer service and merchandise, sale, inventory and warehouse management. While all of these aspects are important for retail operations, in this work, we focus attention on visual merchandising and, in particular, on the planogram integrity and compliance [2]. The aim of visual merchandising is to drive, attract and motivate a consumer to purchase a particular product. In a retail store, the planogram is often extensively used as a visual merchandising tool. The planogram is a detailed visual map of the products in the store indicating the position of the products in order to supply their best location for suppliers. The planogram attempts to capture the absolute physical positions of an assortment, the relative locations of products in an assortment, the amount of space allocated to each category and each type of stocking keeping unit (SKU) within the category. In other words, the planogram is designed for reasons, such as increasing sales and profits, introducing a new item, supporting a new merchandising approach, *etc*. Deviating from the planogram defeats the purpose of any of these goals.

A study of the National Association for Retailing Merchandising Services (NARMS) found that a 100% planogram compliance after an initial reset, within two weeks, yields a sale lift of 7.8% and a profit improvement of 8.1% [3,4]. A fundamental aspect is the development of a shelf planogram to reflect the real need of the product in that particular location and in that time frame. Compliance with the planogram is crucial to avoid stock-outs and to maintain the expected level of sell-out; an estimate indicates that 10% of planogram errors leads to an increase of 1% in stock-outs and, consequently, decreases the sell-out by 0.5%. An effective and functional planogram will be most successful when conducted on objective physical findings and not just with the policies of promotional products. Identifying out-of-stock (OOS) with certainty and in real time and developing a functional planogram are therefore related and equally important needs.

In retail stores, a planogram can concern the entire map of the store, some aisles, some shelves or a specific category of products. Among its multiple aims, the planogram is used for product placement, marketing decisions and for customer experience, so that the correct layout of a product can increase brand loyalty and, consequently, costumer satisfaction [5]. Currently, there are several software platforms for designing a planogram in 3D, but the real problem is not related to the generation of a planogram, rather to the planogram integrity and compliance. Planogram compliance refers to the merchandising management and also to the supply chain [6].

In this work, to protect the integrity of the planogram, we propose an embedded system, mainly based on a smart camera, which has been already installed in two different retail stores. The smart camera, described in detail in the following section, is installed at points considered strategic for the stores taken into consideration. Each embedded system produces an amount of information useful not only to assess the integrity of the planogram, but also concerning out-of-stock.

In the literature, there are many studies working on the analysis of planograms, e.g., [3,5,7]. In particular, they demonstrate that planogram maintenance is a key aspect to increase shelf value and to improve sell out. A series of patents for the determination of inventory conditions, the determination of product display parameters, the planogram extraction and the detection of stock out conditions based on image processing can also be found in [8,9,10,11]. At the base of these products, there are algorithms able to detect and extract several features, such as logos [12] or books [13]. In [14,15], it is possible to retrieve examples of software that use images for automatic planogram compliance and generation. Both are commercial products that are based on images manually collected in front of the shelf and analyzed on a web-based platform or in a local desktop application. Differently from our system, they are not real time, and also, they are always managed by a person using a camera; for this reason, they are not monitoring the planogram continuously. This aspect is important not only to continuously verify the planogram maintenance, but it is also essential for the shelf-out-of-stock (SOOS) management system based on real-time sensor measurements and for the costumer’s activity recognition for the shelf interaction. In fact, the same system architecture has been extensively used to monitor both the behavior of the costumers in front of a shelf [16,17,18,19,20] and the absence of products on the shelves [21,22]. Besides, thanks to its modularity, this system can be applied to analyze the human behavior, both in the retail stores and in applications concerning intelligent environments, such as ambient assisted living applications. The above three issues (planogram maintenance, SOOS management and costumer’s activity recognition) represent the most important challenges for retailing, since they are strictly related to the satisfaction of customers [23,24].

The embedded system here proposed integrates both the camera and the software for image processing and the computation of differences all in a low cost architecture (of about 200 dollars), and it has a return on investment (ROI) of six months. With respect to the state of the art, the system is battery based and very easy to install and use as a tool to provide a diagnostic measure over a finite time period (e.g., two weeks) and then to define a policy according to the store staff. By collecting data from multiple shelves and from different stores receiving only synthetic data (*i.e*., the percentage of planogram compliance), the cloud-based architecture of the system is a crucial aspect to perform planogram analysis in geographically-distributed retail environments. To our knowledge, the proposed solution is the only affordable and scalable solution available in this field: it provides an easy to install, low cost, scalable and affordable solution to the problem of planogram maintenance, both from the hardware and software point of view. Moreover, the system gives, over an Internet of Things (IoT) cloud-based architecture, many additional data that do not concern the planogram, e.g., out-of-shelf events promptly notified through SMS and/or mail, thus opening future works and improvements of the system on the more general aspect of shelf knowledge and understanding.

In summary, the proposed work introduces at least the following three novel aspects:

- The embedded sensor: the battery-based camera is a new design that is very specialized for the purpose described here, and it is a quite unique solution on the market with a really strong emphasis on power consumption; image processing procedures, data transmission and representation are totally focused on the general design of having a low cost, highly scalable, high resolution, battery-based smart vision system.
- The cloud-based data infrastructure: the retail industry is intrinsically distributed and scalable (store chains have hundreds of stores placed all over the world with tens of shelves each); to our knowledge, this is the first cloud-based system that considers the shelf and its planogram as a part of the IoT world in order to: extract data from a smart sensor, share it, inform end users and perform deep learning on collected data to train the system to learn new information and to autonomously provide solutions to problems.
- The application: automatic planogram inspection is a really novel application in the scenario of IoT; it will ensure a strong impact on the grocery and retail market by bringing sell-out improvements and advancing the store management state of the art. Our proposed architecture automatically verifies the compliance of the planogram by simply taking a snapshot of the shelf. Then, the system provides information of incorrect displacements by means of a software GUI (see the Results Section). A periodic intervention of the staff is necessary, usually at opening and/or closing time, since the project is not focused on automatic replenishment, rather on automatic detection using low cost, battery-based sensors. Therefore, the adjective “automatic” mainly refers to the change detection approach, performed using only a minimum amount of *a priori* knowledge (the correct planogram or “first” snapshot). Besides, the system provides a fully-automated measure of the correct product displacement in terms of the percentage of planogram compliance.

The paper is organized as follows: The next section provides a description of the proposed embedded system with a particular focus on the camera system, interfaces and computational aspects of the multimedia sensor network. Section 3 is focused on the image processing module, while Section 4 gives details about the actual high level software architecture. The results of large-scale experiments and a discussion are provided in the last two sections.

## 2. Smart Camera Description

The purpose of this work is to provide a system, mainly based on a smart camera, able to acquire and to analyze significant parameters of a retail store in order to detect differences from an accepted situation, *i.e.*, an approved planogram. This is a complex task, so it is essential to propose an innovative solution that allows concentrating all of the surveys and the necessary measures on a limited number of devices. Moreover, these devices must be for simple and immediate installation and in communication. The main idea is to easily and quickly install these smart cameras by using the lowest possible amount of connecting cables and by building the camera as a modular structure. These features allow optimizing both the cost of the camera and the time and costs for installing the system.

The embedded system design here proposed is also based on the vision that distributed embedded vision sensors are a proper solution to cope with the automation of the planogram maintenance process. During the last few years, this area attracted more interest due to different visions and planning approaches. This last concerned new or enhanced location and context-aware, self-configuring and self-healing applications based on collaborative signal and information processing in wireless networks of computing sensor nodes embedded or placed, almost everywhere, in the environment. Our system goes in this direction, and for the state of the art of intelligent retail environments, to our knowledge, it is the first solution that provides a comprehensive architecture (hardware, distributed image processing and cloud-based data management) for planogram maintenance. Being a network of cooperating embedded systems, the proposed solution is also able to deal with many different types of constraint’s design: low power consumption and long life battery supply, a low cost solution with high scalability (typical of the retail market) and small-sized devices that are easy to install. Cooperation among autonomous nodes to solve a common goal is the best possibility. The nodes in the network contain a combination of sensor, processing and communication devices. Connections between nodes and networks are wireless and self-organizing.

The application of this project allows the realization of highly integrated, economic, complete and easy to use systems for a large number of users. In the following subsections, the proposed system, which can be also used in environments other than retail stores, as, for example, in ambient assisted living, is described in more detail.

### 2.1. Definition of Technical Specifications and Modularity

According to the main specifications, the aim is to realize a smart camera with the following characteristics:

- small size;
- battery supply;
- low battery consumption (>6 months of operations);
- high resolution images;
- ability to capture images in the infrared frequency (not essential for the purpose of this work);
- transmission via Wi-Fi of information acquired;
- interface to connect other additional sensors;
- modularity.

The small size requirement was satisfied by using both specific components able to perform multiple functions simultaneously and multilayer printed circuit boards. From a preliminary feasibility study, it was derived that most smart cameras have frontal dimensions of 36 × 36 mm, and in any case, they are not expected to exceed a size of 40 × 40 mm. Each module that composes the smart camera provides an independent power input handled by the master module, so that it can be activated only when needed. The master unit management should provide an ultra low power mode. The smart camera can be powered either by an external power supply or with battery power. Batteries must ensure a cycle life of at least six months. Considering that nowadays, in almost all environments, there is a Wi-Fi router for Internet use, we choose to use Wi-Fi as the wireless system for data transmission, which, in addition to enabling an easy installation, provides high speed communication at 54 Mbps and beyond. Since not all measures, such as humidity, may be detected by the camera, to make the smart camera an open device adaptable to future needs, we included a serial-type external communication interface with the inter-integrated circuit (IIC) standard to link external devices.

**Figure 1:**
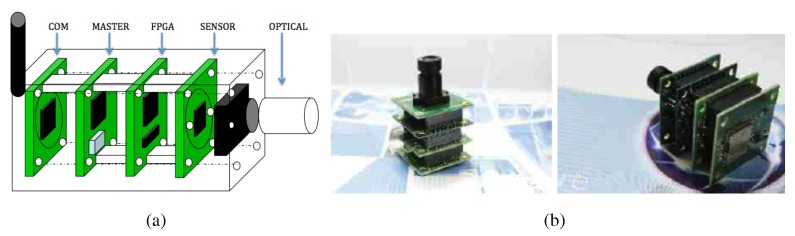
(**a**) Illustrative scheme; (**b**) Two views of the real camera. The smart camera.

Figure 1 shows an illustrative scheme (Figure 1a), which highlights the five different components, and two different views (Figure 1b) of the smart camera.

Modularity is necessary to allow configuring the smart camera according to the real needs of each specific environment to be monitored. In this way, we will be able to optimize the costs of the whole system.

**Figure 2:**
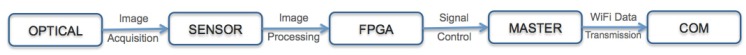
Diagram of the five components with their input/output connections.

Figure 2 makes explicit the communication among the smart camera modules. Components are strictly connected together. Modules are separated mainly for design and revision simplification. An important characteristic of the proposed architecture is to be able to easily remove or upgrade a single module without the need of a complete redesign (*i.e*., a new vision sensor, a communication protocol different from Wi-Fi, a new FPGA, and so on). Each of the five modules is described in detail in Table 1, considering also its technical specifications.

**Table 1:** Description of each module of the smart camera.

NAME	DESCRIPTION and TECHNICAL SPECIFICATIONS
**COM**	Wi-Fi data transmission module (COM): Wi-Fi standard IEEE 802.11 b/g Texas Instruments Model CC3000; embedded IPv4 TCP/IP stack; radio performance TX power: +18.0 dBm at 11 Mbps, CCK; low-cost and low-power.
**MASTER**	Master module managing the feeding of each module and the IIC interface for external devices, as well as connections to the battery, to external power and to another smart camera to achieve stereoscopic vision (MASTER): Micro controller Cortex M0+ Freescale Model MKL25Z128VLK4 with a 32-bit ARM Cortex-M0+ core running at 48 MHz; the energy-saving architecture is optimized for low power with 90-nm TFStechnology, clock and power gating techniques and a zero wait state flash memory controller.
**FPGA**	Image processing module (FPGA): Lattice Model LFXP2-5E-5FTN256C; number Of macrocells: 5000; maximum operating frequency: 200 MHz; number of programmable I/Os: 172; data RAM size: 10 KB; supply voltage: 1.14 V; supply current: 17 mA.
**SENSOR**	Management module of the optical sensor that acquires and processes images detected by the sensor (SENSOR). The ON Semiconductor MT9P031 is a 1/2.5-inch CMOS active-pixel digital image sensor with an active imaging pixel array of 2592H × 1944V. It incorporates sophisticated camera functions on-chip, such as windowing, column and row skip mode, as well as snapshot mode. It is programmable through a simple two-wire serial interface. The 5-Mp CMOS image sensor features ON Semiconductor’s breakthrough low-noise CMOS imaging technology that achieves CCD image quality (based on signal-to-noise ratio and low-light sensitivity) while maintaining the inherent size, cost and integration advantages of CMOS. Other features: High frame rate Superior low-light performance Low dark current Global reset release, which starts the exposure of all rows simultaneously Bulb exposure mode, for arbitrary exposure times Snapshot mode to take frames on demand.
**OPTICAL**	Optical module (OPTICAL): lens with a focal length of 2.5 mm and a maximum resolution of 10 Mpix.

### 2.2. Master Module and Wi-Fi Communication Design

**Figure 3:**
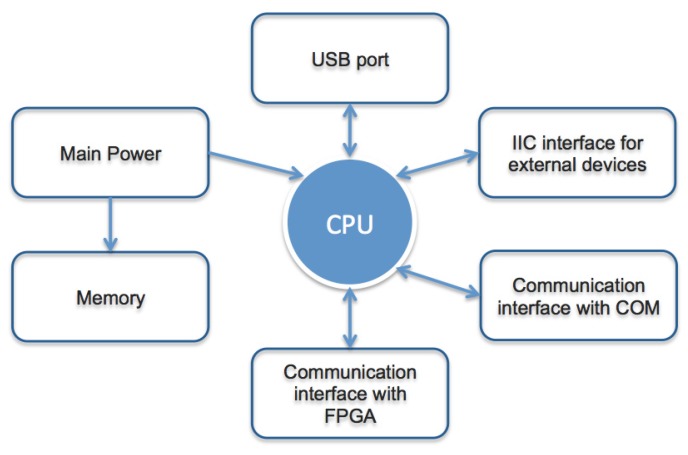
Flowchart of the camera MASTER module.

The aim of the master module is to manage the operation of the smart camera. It consists of the parts shown in the flowchart of Figure 3:

The CPU manages the smart camera function and consists of a Cortex M0+ 32-bit microcontroller. The main power manages the power input from an external power supply and the input of a power supply with a battery charge status control. The memory component compares the captured images to detect possible movements; it contains all possible smart camera configurations and provides also the function for online updating of the firmware. The IIC interface connects external devices. The IIC bus provides the ability to connect multiple devices on the same bus. The communication interface with the Wi-Fi module uses the serial peripheral interface (SPI), providing high speed data transfer. The communication interface with the image acquisition module is the most complex, because it provides an SPI interface for the configuration management and the control of the video module and a 16-bit parallel bus for the acquisition of images. The (Universal Serial Bus) USB port is required to update the firmware and also to read images in memory. Regarding the definition of the Wi-Fi communication module, it is important that it is able to perform all of the transmitting and receiving data functions, *i.e*., it has all of the necessary resources to manage the Wi-Fi protocol and all of the main protocols used in network communications, such as TCP (Transmission Control Protocol), UDP (User Datagram Protocol), IP (Internet Protocol) and HTTP (Hypertext Transfer Protocol).

### 2.3. Image Processing Module

The image processing module, in addition to reading the image detected by the sensor, is able to optimize the sensor parameters and includes memory necessary to save the images. This module consists of the following parts:

- an FPGA to process the acquired images;
- volatile memory for the temporary storage of more images sequentially acquired (buffer);
- flash memory where sample images are stored;
- a voltage stabilizer, managed by the master module, which powers all components.

The FPGA carries out all of the functions necessary to provide the master module with the images acquired by both the video sensor and the infrared sensor. The image processing module is based on a fast, simple and accurate algorithm for change detection that will be detailed in a following section.

**Figure 4:**
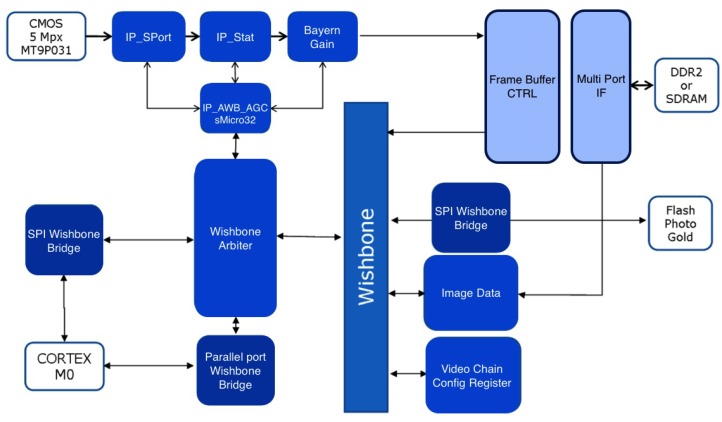
Flowchart of the FPGA operations.

The FPGA model used is the Lfxp2-5e-5ftn256c, which is pin-to-pin compatible with models equipped with higher capacity, necessary to increase the smart camera performances. The images are acquired with a maximum resolution of 5 Mpix at 20 frames per second, and if the resolution decreases, the frame rate can be greatly increased. To manage this huge amount of data, a 16-Mbit parallel bus up to a speed of 100Mhz has been provided. To take advantage of this speed, the image acquisition module must be connected directly on the board with the operating system iMX6 or directly to the network via a 1-Gbit Ethernet port. In this work, we take into account the first case by developing a specific driver for the operating system. Figure 4 describes the sequence of operations of the FPGA. In the following, the single functions are briefly listed:

- IP sport: interface towards the sensor;
- IP core stat: statistics of the image in real time;
- IP Bayer gain: management of gains for each channel;
- IP-AWB-AGC-SMicro32: white balancing and auto gain managed by sMicro32;
- IP multiport and DDRinterface;
- IP SPI: used to communicate with the external FLASH;
- IP parallel port: the parallel port is structured with generic omnidirectional GPIO:
  - DATA PATH 16-bit only in output;
  - CTRL;
  - 1 STROBE: on the rising edge;
  - 1 OE: output enable;
- IP SPI: SPI to communicate with the external Micro in slave mode for access to all registers of the FPGA module/sensor.

## 3. Smart Camera: An Image Processing Approach to Planogram Integrity

**Figure 5:**
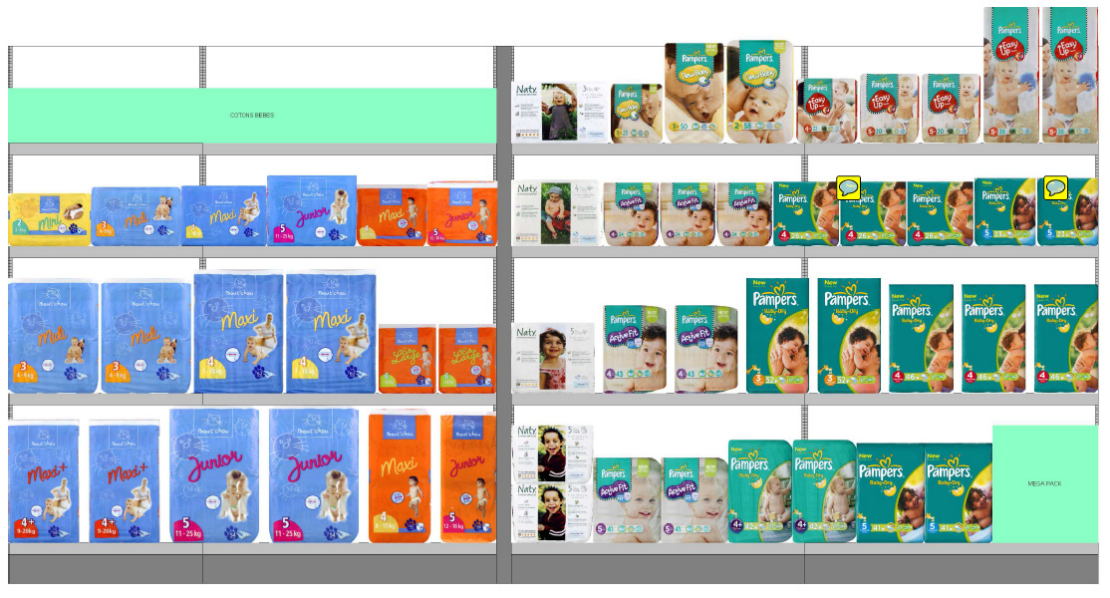
Example of a planogram used in the retail market and described by an XML file. This format is typical of several planogram description software programs available on the market. The most famous is Spacemen, distributed by Nielsen.

Figure 5 shows an example of a frontal image of product positioning. The smart camera described in the previous section provides images to capture the physical location of products on the shelves. The smart camera system knows the planogram, *i.e*., the better position of the products on the shelves. Therefore, to detect planogram integrity, the system automatically matches the approved planogram, stocked on the server, and the pictures from stores. Departing from the image acquired from the acquisition module of the smart camera, the implemented system matches the planogram image (base) with the acquired image and provides a comparison image, calculating the differences between the two images. The algorithm compares the images detecting areas with “big” differences in dimension, by subtracting the corresponding pixel of each image and providing an alert when the difference is greater than a fixed threshold. We are not interested in change detection that involves areas smaller than the dimension of a single product (these “small” differences are considered as noise and deleted).

**Figure 6:**
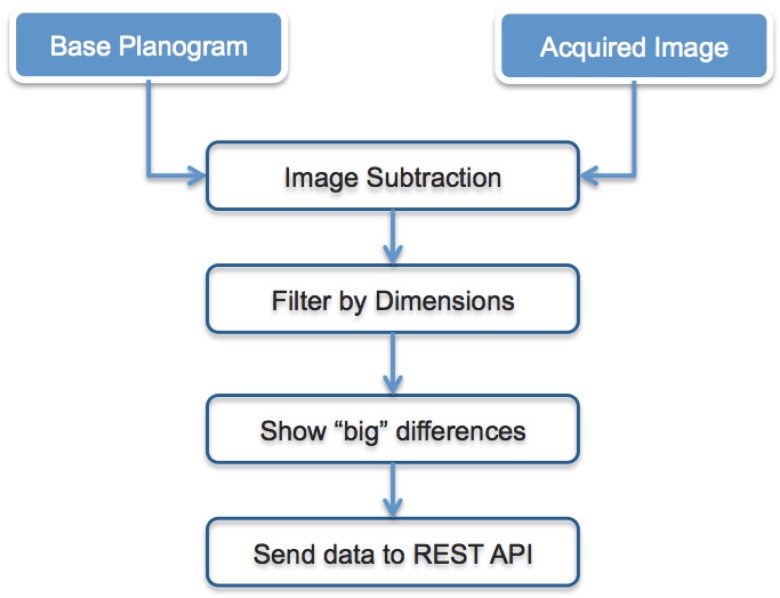
Representation of the implemented image processing algorithm.

**Figure 7:**
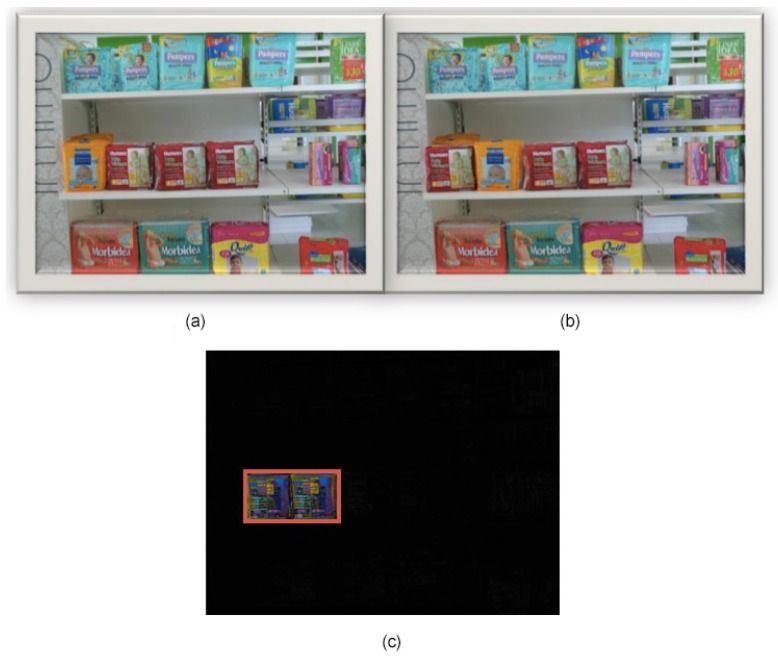
Comparing two planogram images: (**a**) planogram image (base planogram); (**b**) acquired image (actual image); (**c**) visualization of the difference image.

In Figure 6, the architecture of the implemented software is presented. As an example of the system, we show how it acts when an image is acquired and matched with the accepted planogram. Observing Figure 7, there are two images of the example: Image (a) represents the planogram, while Image (b) is the acquired image, clearly referring to the same scene/shelf at different moments.

Analyzing Figure 6, the algorithm processes two input images: the image representing the accepted planogram (base planogram) and the image acquired by the smart camera at a later time. It has to establish how the actual image is different from the planogram accepted as correct. Image subtraction is the result of this comparison. Each pixel value of the first image is compared to its corresponding pixel value in the second image. If the difference between the two values exceeds the fixed threshold, the pixel is represented as the color difference between the planogram pixel and the reference pixel, while if the difference is less than the threshold, the pixel in the difference image is black, as Figure 7c shows. Before saving the difference image, the output of the image subtraction module is processed by the filter-by-dimensions module, which eliminates, that is puts to black, pixels associated with noise. Then the Show—“Big”—differences module represents the difference image. Observing the images in Figure 7, we can see that, in the second image, the planogram has not been completely respected, since there are items in the wrong positions. The algorithm detects the problem and signals the differences by reporting the vertex coordinates of the bounding box of the detected area (shown in Figure 7c by red lines). Only this geometric information is stored in the sensor-cloud infrastructure described in the next section and used for the statistic layer. A local threshold, implemented in the smart camera, is used to exclude small areas and can be manually configured according to the dimensions of the smallest product on the shelf.

The non-black pixels in Figure 7c represent products that in the acquired image are not in the correct position with respect to the planogram image. As we said, this situation will be promptly notified through an alert. If the difference image is completely black, there is a situation of planogram integrity. In a real configuration, the arrangement of products in the shelf could not be so ordered as in Figure 7. On the contrary, the recognition of multiple instances could be interfered for many reasons: bad illumination, bad positioning, rotation, translation and, in particular, objects being partially occluded. Obviously, in these situations with many differences signaled, the system results in being less useful than in cases with only a few differences, because it requires the robust, supervised intervention of the staff.

## 4. Sensor Cloud Architecture

The web-based architecture can be described as a sensor-cloud infrastructure. It has been evolved and proposed by several IT people in present day [25,26]. The sensor-cloud infrastructure is the extended form of cloud computing to manage sensors that are scattered throughout the network. Due to the increasing demand of sensor network applications and their support in cloud computing for a number of services, the sensor-cloud service architecture is introduced as an integration of cloud computing into a wireless sensor network (WSN) to innovate a number of other new services. Services are mainly devoted to data management and camera configuration, e.g., sending data and receiving acknowledgments of download configuration parameters for every camera.

**Figure 8:**
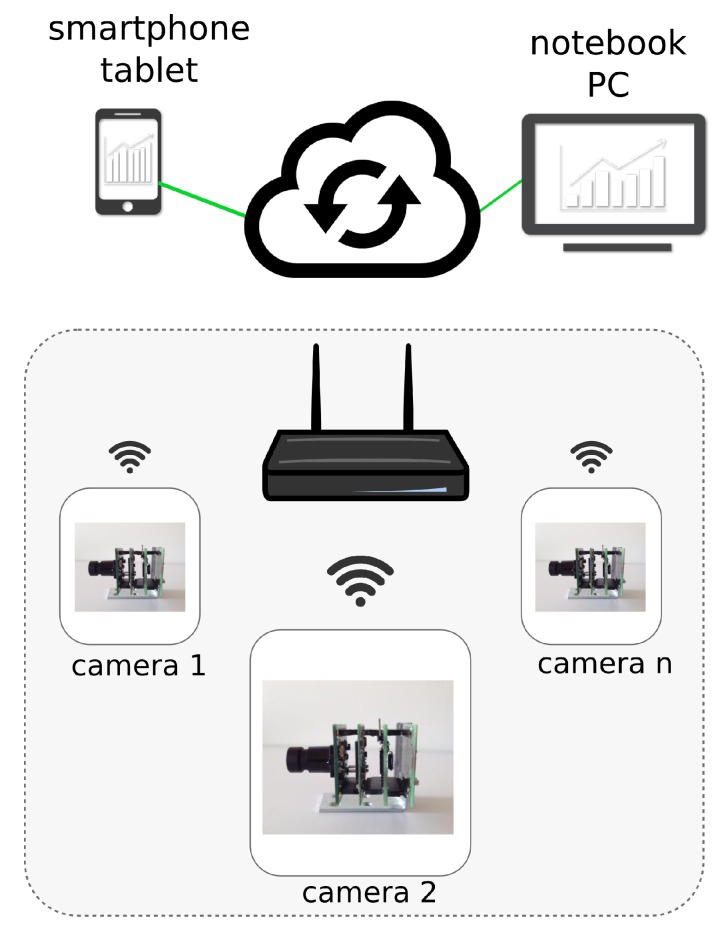
The sensor cloud architecture.

Figure 8 shows a representation of the sensor-cloud infrastructure. Basically, every camera is a sensor node that transmits synthetic raw textual data to the cloud over the Wi-Fi connection. Therefore, each camera communicates with the cloud architecture by means of Wi-Fi transmission data. Data can be processed through different devices (smartphone, tablet or notebook).

When a WSN is integrated with a cloud computing environment, several complexities, like the storage capacity of data collected on sensor nodes and the processing of these data together, would become much easier. Since cloud computing provides huge storage capacity and processing capabilities, it enables collecting a huge amount of sensor data by linking the WSN and cloud through the gateways on both sides, that is the sensor gateway and the cloud gateway. The sensor gateway collects information from the sensor nodes of the WSN, compresses and transmits it back to the cloud gateway, which, in turn, decompresses and stores it in a sufficiently large cloud storage server. In the proposed application, every node (smart camera) is a sensor able to send synthetic data to the cloud. The cloud-based web application allows one to:

- define the region of interest (ROI) of the reference image sent by the camera in the first configuration phase;
- define users at different levels to access statistics and to receive alerts (via mailor SMS);
- store data from every node into a database to allow detailed analytic reports;
- define an alert threshold as a maximum level of planogram errors;
- send alerts via mailor SMS when the alert level of the planogram errors is reached;
- compare data coming from different categories/different stores.

All of the software is provided as a service and is fully developed in Php language, using a MySQL DBMS. The reporting system is based on Spago BI. An example of the analytic report interface is reported in the Results Section.

In this section, we also introduce a linked data-driven and service-oriented architecture to address the issues discussed above. The main motivation is the need for a multi-camera, multi-store policy on a cloud-based data infrastructure that can allow one to manage and enrich planogram maintenance data able to give a decision support system to final users and their stakeholders (retailers, brands and visual merchandisers).

The four major contributions of this section are:

1. Linked data-principles are applied to model and expose the metadata of both planogram resources and planogram compliance analysis. Web services and APIs are provided for that. In this way, not only resources are connected, but also services’ description and resources are exposed in a standardized and accessible way in the retail data scenario.
2. Existing heterogeneous and distributed planogram repositories, *i.e*., brand and retailers’ web interfaces (services), are integrated on the fly by reasoning and processing of linked data-based service semantics for planogram description and integration.
3. Metadata retrieved from heterogeneous web repositories, for instance Nielsen Spaceman Suite (NSS) resource metadata [27], are automatically lifted into RDFand exposed as linked data principles, enriched with planogram compliance details.
4. A set of RESTful APIs is developed on top of the proposed framework to allow third party applications to consume and interact with the data exposed by our approach.

## 5. Results

**Figure 9:**
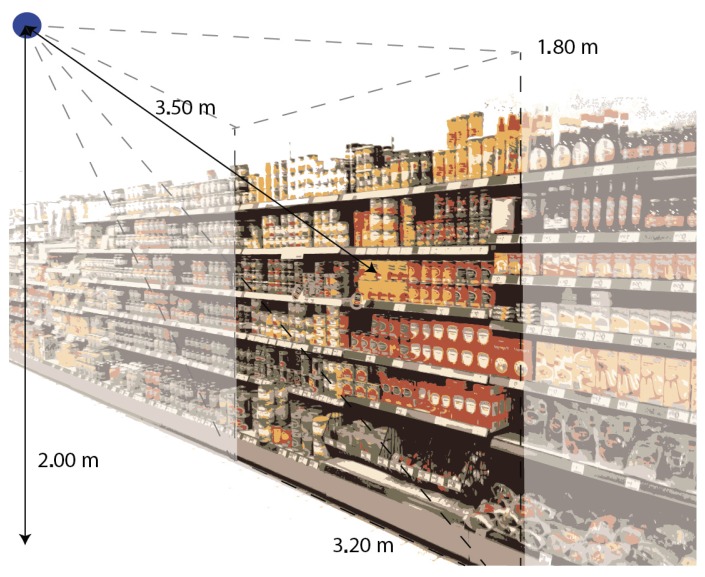
Representation of the experimental setup.

In the experimental phase, the camera was fixed at two meters above the floor at a distance of 3.5 m with respect to the center of the shelf. The maximum visualization area was 1.8 m × 3.2 m, wider than the height of the shelf (1.5 m). This experimental setup is shown in Figure 9. The camera is in a fixed central position, and in this experimental phase, we have not tested the performances of the system by rotating or translating the camera. In any case, we are really confident that the system is not affected by small 3D deformations, because the basic idea of the proposed approach is to work on rectified images and change detection, with the purpose of identifying non-correct product placement from a pre-defined position. Tests were performed in two real stores in Italy, both on the diaper shelves. The computational time to elaborate differences between 1280 × 960 images is about 760 ms.

We measured the power consumption of each board in idle state (Pidle) and at full load (Pmax), *i.e*., with the radio transmitting, the camera acquisition and the algorithm running. For the shelf monitoring application here described, it is reasonable to assume that, with a monitoring period of one minute every day, the smart camera powered by two C batteries (Long-Life Alkaline, Size C, 1.5 V, 7000 mAh) will last for about one year. Indeed, the camera and the algorithm can run just once a day, usually just before the store opening time; therefore, the board will be on for less than two minutes every day (considering a frame rate of one frame per day, the start-up time, the elaboration time and the data transmission via Wi-Fi). The start-up time of the system is 500 ms, and the processing time of a full pixel screen shot is about 3 s, depending on the amount of differences between the current image and the reference image; the transmission of synthetic processed data takes about 500 ms (raw text data). The resulting duty cycle is equal to 5%, and supposing a battery capacity of 7000 mAh, the multimedia node life is more than the expected one year. This is the actual parameterization of our system, but the user can choose a different time interval. For the planogram application, the information acquired is realistic, because planogram maintenance activities are usually performed early in the morning. In any case, there are no technical limitations on this side, apart from that power consumption is affected linearly by this parameter. Table 2 reports different consumption tests to prove the efficiency of the actual choice. The duration of the battery results is inversely proportional to the resolution of the image. We took into account only image resolution for two main reasons: (i) time parameters are not very important, because our system takes only one snapshot of the shelf a day; (ii) the performance of the system in verifying the integrity of the planogram strongly depends on the resolution (the higher the resolution, the more precise is the comparison between the base planogram and the acquired image).

**Table 2:** Battery-based life time *vs*. image resolution.

Solution	Resolution	Time (days)
**Actual**	1280 × 960	360
**High Resolution**	2560 × 1920	60
**Low Resolution**	800 × 600	900

Real data were essential to test the overall behavior of the system with regard to different lighting conditions (even if the store is an indoor environment, the external light causes a deep increase of light during day or night), occlusions (people passing by), store people behaviors (refill team working on the area, wrong product positioning). The system installed runs for 25 days collecting a huge amount of data and different events on planogram maintenance. In particular, the total amount of pictures processed was about 1000. In this period, planogram errors were also monitored manually, day-by-day, to have a ground truth able to evaluate the results of the automated process. Therefore, the ground truth was validated using human inspection in the real scenario. Incorrect product placements were measured by evaluating manually the differences between day-by-day measurements. Comparing the manual annotations with the results of the system, we reached 96% reliability with respect to the ground truth.

**Figure 10:**
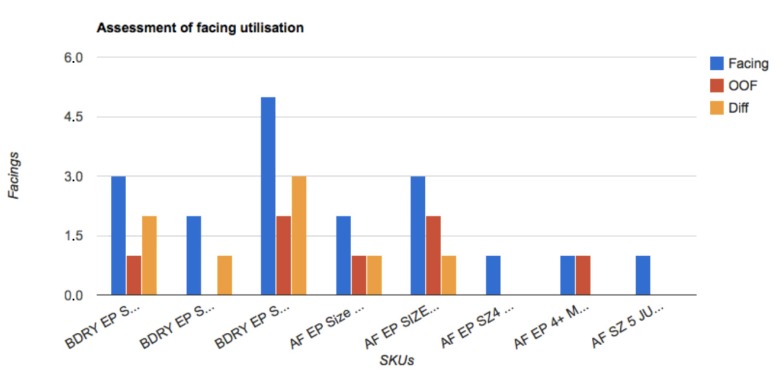
Comparison between the base planogram and current out of facing (OOF) of products for different stocking keeping units (SKUs) on the shelf. Differences (Diff) can be useful for planogram optimization based on optimal space allocation.

The system provides reports showing a comparison between the planogram and the real usage of the products on the shelf. This provides several new ideas on the way to improve the planogram’s profitability [28] for a typology of store that sells a specific category of products: motor vehicles, electronic equipment, chemicals, toys, household products (detergents, diapers, cosmetics), and so on. In the experimental phase, we consider a specific store of diapers as the dataset, as Figure 10 shows. The yellow bars in Figure 10 highlight the presence or not of a pack of diapers (characterized by brand, size, colors, and so on) on the shelf, by comparing the base planogram with the image acquired by the camera. This comparison is useful not only to detect differences from the base planogram, but also to prevent shelf-out-of-shelf eventually. The product is not in the correct position because it may have been moved with respect to the position of the base planogram or it may be out (shelf-out-of-stock problem).

Therefore, in Figure 10, blue bars show the number of facings on the current planogram; red bars show the real number of facing utilization based on real-time data coming from shelf detector sensors [22]. Yellow bars are the differences between the previous two and, basically, suggest which kind of implementation should be applied, not only for the planogram integrity, but also to improve the planogram. Every camera sends its data to the cloud server using the REST API provided by the proposed data architecture. Here, we describe the standard data coming from a single camera: Every message contains the total compliance percentage ($total_{err}$) measured as the ratio between wrong placements and total measured area, in pixels. Several bounding boxes indicated by $x_{1}$, $x_{2}$, $y_{1}$, $y_{2}$ and an identification value also describe every wrong placement area. Every camera is linked to the store and is also described by a configuration file that gives to the system information about the geometry of the camera installation, the IP of the gateway, and so on. Using the proposed data architecture, the brands, retailers and visual merchandisers can extract comparative data between categories or stores, can use data to link them to other sources and to obtain useful insights and can apply premiums or penalties to stores that have great or bad planogram maintenance performances. An example of planogram optimization insights is reported in Listing 1, where, first of all, the identification number of the store is indicated and, then, the parameters of the camera, as previously described.

## 6. Conclusions

In a retail store, the use of a planogram is important for different aspects: increasing sales, increasing profits, introducing a new item, supporting a new merchandising approach and better managing the shelves. The planogram is often used in retail operations as a means of visual merchandising. A problem related to the planogram is maintaining its integrity: real-time integrity is a crucial problem for vendors. The respect of the position of products on an accepted planogram implies attracting and motivating a costumer to purchase a product. The research described in this paper proposes a new embedded system that is highly integrated, economic, complete and easy to use for a large number of users. To evaluate the integrity of the planogram, a smart camera estimates the difference image between the base planogram and reference images. We also reported preliminary results as regards the planogram integrity. The results are efficient, since the system in real time provides information concerning both the deviation from the planogram and out-of-shelf events. The actual solution proposed in this paper is a really smart solution, battery-based (not common for visual sensors) and able to automatically perform planogram maintenance measurements with precise feedback from users. The solution is also low cost and provides an industrial solution with high scalability for retailers. The data solution, cloud based, but with distributed processing, is also very inexpensive and simple. There are no particular limitations, and the solution, developed together with a company working in retail technologies, seems to be a nice Columbus’s egg. The main improvements can be done in the design of a plastic box and on the strong optimization of power consumption, to be able to have a two-year battery life, maintaining low cost and small dimension constraints.

Listing 1 **Example of a JSON schema.**


{
      "store": {
             "id": "1"
      },
      "camera": {
             "id": "99",
             "total_err": [
                    {
                          "perc": "12",
                          "time": "2012-04-23T18:25:43.511Z"
                    }
             ],
             "areas": [
                    {
                          "ida": "1",
                          "x_1": "11",
                          "x_2": "29",
                          "y_1": "141",
                          "y_2": "173",
                    },
                    {
                          "ida": "2",
                          "x_1": "123",
                          "x_2": "229",
                          "y_1": "1041",
                          "y_2": "1096",
                    }
             ]
      }
}
